# The Augusta Meeting of the Medical Association of Georgia

**Published:** 1906-06

**Authors:** 


### THE AUGUSTA MEETING OF THE MEDICAL ASSOCIATION OF GEORGIA.

THE fifty-seventh annual meeting of the Medical Association of Georgia was held in Augusta, April 18, 19, 20. The attendance was large, and the scinetific sessions were greatly enjoyed.

The Association showed a large increase in membership, due to the great activity in the organization of county medical societies, and the efficient work of the Secretary. The additional work laid upon the Secretary and Treasurer by the increase in membership warranted raising the salary of that officer from $500 to $1,000 a year. Important results of the meeting were the following resolutions, which were adopted:

*Resolved,* That it is the sense of this Association that the minimum fee for life insurance examinations should be $5.

WHEREAS, It has been brought to the attention of this body that there is a pure food bill at present before the Congress of the United States, known as the Hepburn bill; and

WHEREAS, This bill meets with the approbation of this body representing the medical profession of Georgia; therefore, be it

*Resolved,* That this body earnestly requests the Senators and Representatives in Congress from the State of Georgia to vote for the said bill, and that they use all their influence to further its passage.

Savannah was chosen as the place of meeting in April, 1907. The annual election of officers resulted as follows: President, H. H. Martin, Savannah; Vice-president, T. E. Oertel and J. W. Palmer. Dr. L. H. Jones was re-elected Secretary-Treasurer.

Drs. J. D. Coleman, of Augusta; H. F. Harris, of Atlanta, and George R. White, of Savannah, were elected delegates to the American Medical Association.

---

THE JOURNAL-RECORD desires to say that in controversial matters which are at issue at the present time, it shall be its policy to maintain a position of neutrality. Its columns are open to all who wish to express their convictions regardless of harmony with the personal views of the editor.

---

DANIEL DAVID QUILLIAN, M.D., Atlanta, graduate of the Atlanta Medical College; a member of the American Medical Association and the Medical Association of Georgia; for seven years sanitary inspector and attending physician of the Georgia State Normal School, Athens; one of the most prominent physicians of Clark County, Ga., died at his home in Athens, April 17, from pneumonia, after an illness of one week.

